# Unfavorable microbiological impact of directly duodenal biliary drainage in patients with perihilar obstruction: a preliminary report

**DOI:** 10.3389/fsurg.2025.1538676

**Published:** 2025-07-10

**Authors:** Veronika Rozhkova, Kamuran Tutuş, Selda Kömeç, Erdem Kınacı, Özgür Bostancı, İlgin Özden

**Affiliations:** ^1^ Department of General Surgery, Liver Transplantation & Hepatopancreatobiliary Surgery Unit, Başakşehir Çam & Sakura City Hospital, Istanbul, Türkiye; ^2^ Department of Clinical Microbiology, Başakşehir Çam & Sakura City Hospital, Istanbul, Türkiye

**Keywords:** bactibilia, biliary drainage, carbapenem-resistant Enterobacterales, extensively drug-resistant, multidrug-resistant, perihilar

## Abstract

**Background:**

Biliary drainage is frequently used in patients with perihilar obstruction. This study was designed to compare the microbiological characteristics of patients whose biliary trees were either exposed or not exposed to duodenal fluid, depending on the drainage method used.

**Methods:**

The charts of 71 patients with perihilar obstruction (any etiology causing an obstruction parallel to that of a proximal cholangiocarcinoma according to the Bismuth–Corlette classification) were evaluated retrospectively. The contacted group comprised 20 patients who underwent either endoscopic stenting or percutaneous transhepatic biliary drainage (PTBD) with duodenal extension, while the non-contacted group consisted of 51 patients with either external PTBD or surgery upfront.

**Results:**

Positive bile culture results were identified in 19/20 (95%) vs. 17/51 (33%) patients (*p* = 0.00001) and multimicrobial growth in 13/19 (68%) vs. 4/17 (24%) (*p* = 0.007) patients in the contacted group and non-contacted group, respectively. Colonization of bile with multidrug-resistant (MDR) and extensively drug-resistant (XDR) bacteria was worse in the contacted group: 13/19 (68%) vs. 5/17 (29%) (*p* = 0.02). Significant differences were also found in the frequencies of carbapenem-resistant Enterobacterales (CRE) colonization: in the contacted group, positive CRE culture (*p* = 0.033) and PCR (*p* = 0.01) were more frequent.

**Conclusions:**

The mode of the biliary drainage—duodenal vs. directly external—significantly modifies the microbiological characteristics of the patients with perihilar obstruction. Catheterization methods that entail continuous exposure of the biliary tree to duodenal fluid are associated with higher frequencies of bactibilia, presence of MDR and XDR bacteria in the bile, and intestinal colonization with CRE.

## Introduction

Biliary drainage is frequently used in patients with perihilar obstruction to improve liver function, treat cholangitis, and gain time for additional investigations. Each method of drainage—endoscopic stenting, percutaneous transhepatic biliary drainage (PTBD), or nasobiliary drainage (NBD)—has its advantages and drawbacks; the choice depends on institutional preferences and each clinical situation (1–6). Our recent experience of patients with perihilar obstruction, referred to us after endoscopic stenting and PTBD extended to the duodenum, revealed that these patients tend to have higher frequencies of bactibilia and colonization with multidrug-resistant organisms. This study was designed to document this problem and formulate approaches for its prevention by comparing microbiological characteristics of patients with perihilar obstruction whose bile was and was not exposed to duodenal fluid.

## Method

Charts of the patients treated for perihilar obstruction between October 2020 and May 2023 were retrospectively evaluated. Patients with the following characteristics were excluded: endoscopic or percutaneous drainage installed for more than 2 months, intrahepatic abscesses, and previous hepaticojejunostomy. The charts of 71 patients with perihilar obstruction (any etiology causing an obstruction parallel to that of a proximal cholangiocarcinoma according to the Bismuth–Corlette classification) were included in the study (7). Benign conditions causing a similar level and pattern of obstruction were also included (i.e., iatrogenic biliary stricture, choledocholithiasis, eosinophilic cholangitis).

The type of obstruction was determined using MRI and magnetic resonance cholangiopancreatography (MRCP) with liver-specific contrast agent (gadoxetate disodium).

Patients were divided into two groups based on whether there was communication between the duodenum and the biliary tree. A total of 20 patients were included in the contacted group: 19 underwent endoscopic retrograde cholangiopancreatography (ERCP) and stenting in the referring institution and one patient underwent PTBD with extension to the duodenum. The non-contacted group consisted of 51 patients: 50 patients who underwent external PTBD at our institution or elsewhere and one non-jaundiced patient who underwent upfront surgery (her intraoperative bile sample results were included in the study).

All patients who were referred to us after ERCP and stenting underwent endoscopic stent removal and conversion to PTBD according to the institutional protocol. Bile samples were collected after percutaneous transhepatic biliary drainage or at first admission to our center from previously installed catheters, while maintaining sterility to prevent cross-contamination.

### Biliary drainage

The procedure was performed in the Department of Interventional Radiology under local anesthesia. The approach was chosen based on anatomical consideration, with preferred left-sided approach. Percutaneous biliary drainage was performed with the use of ultrasonography, guidewire, and catheter to gain access to the biliary system. To confirm the area of obstruction, antegrade cholangiography with iodine-based contrast solution was used. Metallic guidewire was applied to pass through to the obstruction area, and a catheter was placed with one end in the distal bile duct.

### Microbiologic investigations

To describe antibiotic resistance patterns of biliary cultures, standardized definitions proposed by a group of experts from the European Centre for Disease Prevention and Control (ECDC) and the Centers for Disease Control and Prevention (CDC) were applied (8). They were created for ESKAPE pathogens—*Staphylococcus aureus*, *Enterococcus* spp., Enterobacteriaceae, *Pseudomonas aeruginosa*, and *Acinetobacter* spp.—the leading cause of nosocomial infections throughout the world (9). If the detected bacteria were beyond this classification, the abovementioned definition was still applied, as the authors suggested (8). Multidrug-resistant (MDR) was defined as non-susceptibility to at least one agent in three or more antimicrobial categories. Extensively drug-resistant (XDR) was defined as non-susceptibility to at least one agent in all but two or fewer antimicrobial categories (i.e., bacterial isolates remain susceptible to only one or two categories). Pandrug-resistant (PDR) was defined as non-susceptibility to all agents in all antimicrobial categories (i.e., no agents tested as susceptible for that organism).

Rectal swab samples were inoculated onto CRE chromogenic agar to identify patients colonized with carbapenem-resistant Enterobacterales (CRE). The CRE PCR (BD Max, Becton Dickinson, USA) is a real-time PCR test that detects OXA-48, VIM/IMP, KPC, and NDM from rectal swabs.

Fluid samples were cultured on 5% sheep blood agar, eosin methylene blue agar, and chocolate agar. The bacteria that grew were identified using matrix-assisted laser desorption/ionization time-of-flight mass spectrometry (MALDI TOF MS, Bruker, USA). Antibiotic susceptibility tests were conducted using the Phoenix M50 (BD, USA) automated system, following the European Committee on Antimicrobial Susceptibility Testing (EUCAST) standards.

### Statistics

Continuous variables were expressed as mean ± standard deviation or median (range) depending on normality of distribution. Student's *t*-test was used to compare normally distributed data and the Mann–Whitney *U* test to compare the data with skewed distribution. Categorical variables were reported as number (*n*) and percentage (%) and compared using chi-square test (*χ*^2^) (with or without Yates correction) and Fisher's exact test. *p*-Values <0.05 were considered statistically significant.

## Results

The primary diagnoses of the patients are presented in Table 1. The two groups were similarly distributed, although there was a non-significant trend toward a higher incidence of perihilar malignancies in the non-contacted group and choledocholithiasis in the contacted group. Preprocedural CRP levels were also higher in the contacted group, although the difference was not statistically significant (*p* = 0.052).

**Table 1:** Patient characteristics.

Parameter	Contacted group (*n* = 20)	Non-contacted group (*n* = 51)	*p*-Value
Age (years)^a^	55 ± 15	57 ± 15	0.26
Sex ratio (M:F)	11:9	32:19	0.846
Diagnosis			
Perihilar cholangiocarcinoma	50% (10/20)	68.5% (35/51)	0.129
Choledocholithiasis	15% (3/20)	4% (2/51)	0.224
Gallbladder cancer	5% (1/20)	6% (3/51)	0.862
Iatrogenic biliary stricture	25% (5/20)	17.5% (9/51)	0.372
Eosinophilic cholangitis	5% (1/20)	0	
Perihilar lymph node metastasis	0	4% (2/51)	
Preoperative laboratory data^b^			
Total bilirubin (mg/dl)	8 (1; 28)	3 (1; 17)	0.88
ALT (U/L)	89 (11; 464)	120 (11; 462)	0.09
AST (U/L)	68 (21; 454)	70 (24; 305)	0.4
CRP (mg/L)	34 (2; 286)	19 (1; 122)	0.052
GGT (U/L)	333 (77; 1,117)	340 (20; 1,481)	0.584

^a^ Mean values (SD).

^b^ Median (range).

The iatrogenic biliary strictures in both the contacted and non-contacted groups were due to postcholecystectomy injuries, with the exception of one patient in the contacted group who had undergone a living donor hepatectomy at another institution.

### Microbiological characteristics of patients

Positive bile culture results were identified in 19/20 (95%) patients in the contacted group and in 17/51 (33%) in the non-contacted group (*p* = 0.00001) (Table 2); 13/19 (68%) and 4/17 (24%) had multimicrobial growth (two and more microorganisms) in the contacted and non-contacted groups, respectively (*p* = 0.007).

**Table 2:** Microbiological data.

Characteristics	Contacted group (*n* = 20)	Non-contacted group (*n* = 51)	*p*-Value
Positive bile cultures	95% (19/20)	33% (17/51)	0.00001
Multimicrobial growth	68% (13/19)	24% (4/17)	0.007
Colonization with multidrug-resistant organisms			
CRE PCR positive	47% (7/15)	13% (4/32)	0.01
CRE culture positive	29% (5/17)	5% (2/40)	0.033

**Figure 1:**
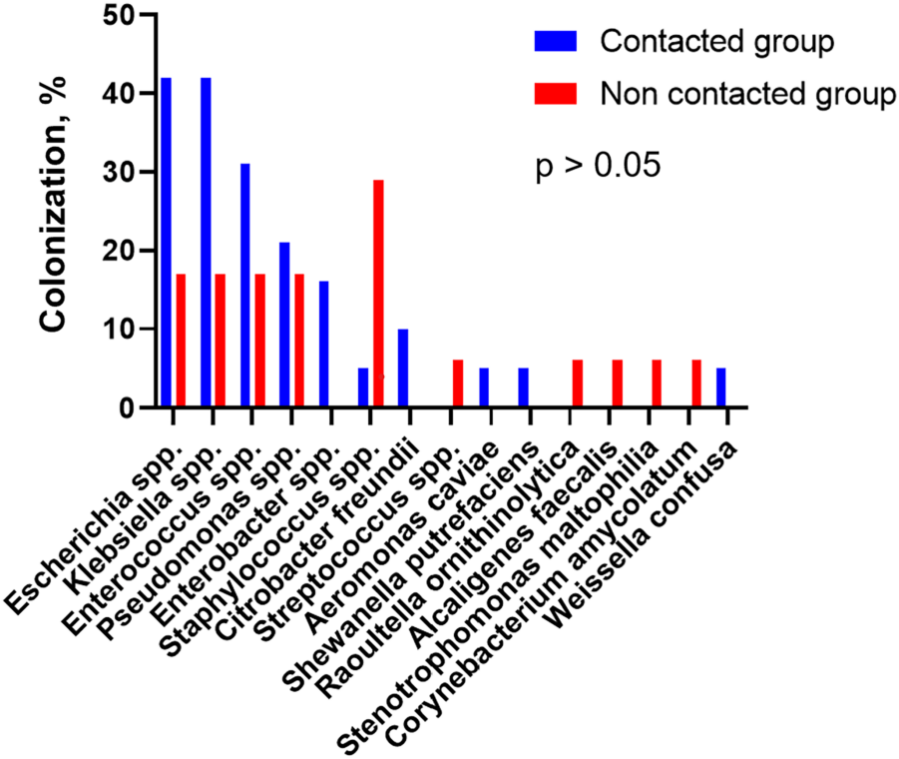
Microbiological colonization of bile.

Table 3 presents the frequency of different organisms in positive bile cultures from both groups. The most commonly cultured gram-negative strains were *Escherichia coli*, *Klebsiella pneumoniae*, and *P. aeruginosa* (Figure 1), and gram-positive strains were *Enterococcus faecalis* and *Enterococcus faecium*.

**Table 3:** Microbiological characteristics of colonized bile specimens.

Bacteria	Contacted group (*n* = 19)	Non-contacted group (*n* = 17)	*p*-Value
Gram-negative			
*Escherichia* spp*.*			0.23
*E. coli*	42% (8/19)	17.5% (3/17)	
*Klebsiella* spp.			0.23
*K. pneumoniae*	36.5% (7/19)	17.5% (3/17)	
*K. aerogenes*	5% (1/19)	0	
*Pseudomonas* spp.			0.87
*P. aeruginosa*	21% (4/19)	17.5% (3/17)	
*Enterobacter* spp.			
*E. kobei*	5% (1/19)	0	
*E. cloacae*	5% (1/19)	0	
*E. hormaechei*	5% (1/19)	0	
Other			
*Citrobacter freundii*	10.5% (2/19)	0	
*Aeromonas caviae*	5% (1/19)	0	
*Shewanella putrefaciens*	5% (1/19)	0	
*Raoultella ornithinolytica*	0	6% (1/17)	
*Alcaligenes faecalis*	0	6% (1/17)	
*Stenotrophomonas maltophilia*	0	6% (1/17)	
Gram-positive			
*Enterococcus* spp.			0.56
*E. faecalis*	21% (4/19)	12% (2/17)	
*E. faecium*	10.5% (2/19)	6% (1/17)	
*Staphylococcus* spp.			0.14
*S. haemolyticus*	0	12% (2/17)	
*S. hominis*	0	12% (2/17)	
*S. aureus*	5% (1/19)	6% (1/17)	
*Streptococcus* spp.			
*S. salivarius*	0	6% (1/17)	
Other			
*Corynebacterium amycolatum*	0	6% (1/17)	
*Weissella confusa*	5% (1/19)	0	

Table 4 describes the antibiotic resistance patterns in both groups of patients. Those who underwent ERCP or internalization of PTBD had higher frequencies of multidrug-resistant bacteria and CRE in bile. MDR and XDR organisms were found in 13/19 (68%) and 5/17 (29%) patients in the contacted and non-contacted groups, respectively (*p* = 0.02). CRE in bile was discovered in 5/19 (26%) and 1/17 (6%) patients in the contacted and non-contacted groups, respectively (*p* = 0.23). All these patients had positive rectal swabs.

**Table 4:** Antibiotic resistance pattern of colonized bile specimens.

Characteristics	Contacted group (*n* = 19)	Non-contacted group (*n* = 17)	*p*-Value
Resistant	68% (13/19)	29% (5/17)	0.02
MDR (multidrug-resistant)	36.5% (7/19)	11.5% (2/17)	0.18
XDR (extensively drug-resistant)	31.5% (6/19)	17.5% (3/17)	0.56
PDR (pandrug-resistant)	0	0	
Carbapenem-resistant Enterobacterales	26% (5/19)	6% (1/17)	0.23

The pattern of carbapenem-resistant Enterobacterales colonization was worse in the contacted group: positive CRE culture and PCR were found in 5/17 (29%) and 7/15 (47%) patients; in the non-contacted group, the corresponding values were 2/40 (5%) and 4/32 (13%) patients, respectively (*p* = 0.033 and *p* =  0.01; Table 2).

## Discussion

Preoperative biliary catheterization and stenting have been implicated as risk factors for postoperative infection through increased frequency of bactibilia (10–13). The original finding of the present study is that in patients with perihilar obstruction the mode of drainage—duodenal vs. directly external—significantly modifies the microbiological characteristics of the patients. To be more specific, patients with perihilar obstruction who have a biliary catheter with a tip in the duodenum have higher bile culture positivity, increased frequencies of biliary MDR bacteria, and gastrointestinal CRE colonization compared with patients whose biliary trees were not exposed to duodenal fluid. There are two possible mechanisms that might explain this observation.

First, it is well established that endoscopic sphincterotomy and stenting causes bactibilia in 83%–100% of patients along with an expansion of the antibiotic resistance spectrum (12–16). Accordingly, for perioperative antibiotic prophylaxis, some centers suggested using different regimens for stented and non-stented cases (12, 13, 15–17). The application of extended spectrum antimicrobics may promote the growth of resistant bacteria and limit available therapeutic choices when a true MDR or XDR bacterial infection arises, forcing reliance on last-line agents. In our contacted group, there were more patients with multi- and extensively drug-resistant microorganisms in bile: 13/19 (68%) vs. 5/17 (29%) in the non-contacted group (*p* = 0.02). Sugawara et al. reported that the presence of MDR in bile culture is associated with surgical site infection (18). Ruzzenente et al. confirmed these data and defined endoscopic sphincterotomy as another risk factor for surgical infectious complications (19). Therefore, it appears that reflux of duodenal fluid, rather than the trajectory of the catheter, is the primary issue. Endoscopic nasobiliary drainage is associated with a lower incidence of postprocedural cholangitis compared to conventional stenting, likely due to specific technical features: infrequent use of sphincterotomy and the absence of catheter side holes in the duodenum (4). To further improve outcomes with sphincterotomy-free techniques, the Nagoya team proposed the use of inside stents – extending from the intrahepatic biliary tree to the common bile duct for tumors at a distance greater than 20 mm above the papilla (20).

Second, in cases of distal obstruction, endoscopic stenting—despite the high incidence of bactibilia— does not cause preoperative complications as long as the stent remains functional. However, these patients still face a risk of postoperative infectious complications (10, 11, 21, 22). In contrast, the three-dimensional nature of perihilar obstruction means that stenting one segment can cause a blockage of the adjacent ducts. When combined with bactibilia, this creates prerequisites for biliary infections. The resulting need for prolonged antibiotic therapy often leads to MDR bacteria colonization (23–26). Hence, stenting has been linked to an increased incidence of cholangitis in patients with perihilar malignancies (2, 3). Similarly, Ba et al. reported that both endoscopic stenting and PTBD extending into the duodenum were associated with higher cholangitis risk (27).

Another potential adverse effect of duodenal communication observed in our study was the increased incidence of carbapenem-resistant Enterobacterales. In the contacted group, CRE culture positivity was found in 5/17 (29%) patients and CRE PCR in 7/15 (47%) patients, compared to 2/40 (5%) and 4/32 (13%) in the non-contacted group (*p* = 0.01 and *p* = 0.033). The global spread of carbapenem-resistant Enterobacterales has led to epidemiological investigations that have identified multiple risk factors associated with its acquisition (28–31). Although inadequate duodenoscope disinfection plays a certain role in CRE outbreaks in some settings, the main risk factor remains the prolonged use of broad-spectrum antibiotics (32–35).

This study has several limitations. Retrospective studies are prone to selection bias. All patients who underwent endoscopic stenting were referred from other institutions after infectious complications or mechanical issues, introducing a potential referral bias. In addition, the indications and technical details of previous ERCP and PTBD were not consistently documented. In some parameters, we failed to achieve statistical significance, likely due to the small sample size. One patient who underwent upfront surgery was also included. The relationship between their preoperative microbiologic data and postoperative complications was not addressed. Furthermore, incomplete medical records led to missing data on rectal swabs and CRE colonization. These limitations may reduce the generalizability of the findings and introduce biases that affect their validity. Future studies with more comprehensive datasets are needed to confirm these findings. Nevertheless, we believe these shortcomings do not undermine the conclusions below.

## Conclusion

The mode of biliary drainage—duodenal vs. directly external—significantly influences the microbiological characteristics of patients with perihilar obstruction. Catheterization techniques that involve continuous exposure of the biliary tree to duodenal fluid are associated with higher rates of bactibilia, increased prevalence of MDR and XDR bacteria in bile, and greater intestinal colonization with CRE. We recommend avoiding drainage methods that maintain ongoing communication between the duodenum and the biliary tree in patients with perihilar obstruction. Biliary drainage without continuous duodenal association are likely to result in a more favorable microbiologic profile and may reduce the incidence of infectious complications.

## Data Availability

The raw data supporting the conclusions of this article will be made available by the authors, without undue reservation.
